# Base Composition and Translational Selection are Insufficient to Explain Codon Usage Bias in Plant Viruses

**DOI:** 10.3390/v5010162

**Published:** 2013-01-15

**Authors:** Daniel J. Cardinale, Kate DeRosa, Siobain Duffy

**Affiliations:** Department of Ecology, Evolution, and Natural Resources, School of Environmental and Biological Sciences, Rutgers, The State University of New Jersey, 14 College Farm Road, New Brunswick, NJ 08901, USA; E-Mails: daniel.j.cardinale@gmail.com (D.C.), klderosa@eden.rutgers.edu (K.D.), duffy@aesop.rutgers.edu (S.D.)

**Keywords:** synonymous codon usage, translational selection, genomic content, mutational bias

## Abstract

Viral codon usage bias may be the product of a number of synergistic or antagonistic factors, including genomic nucleotide composition, translational selection, genomic architecture, and mutational or repair biases. Most studies of viral codon bias evaluate only the relative importance of genomic base composition and translational selection, ignoring other possible factors. We analyzed the codon preferences of ssRNA (luteoviruses and potyviruses) and ssDNA (geminiviruses) plant viruses that infect translationally distinct monocot and dicot hosts. We found that neither genomic base composition nor translational selection satisfactorily explains their codon usage biases. Furthermore, we observed a strong relationship between the codon preferences of viruses in the same family or genus, regardless of host or genomic nucleotide content. Our results suggest that analyzing codon bias as either due to base composition or translational selection is a false dichotomy that obscures the role of other factors. Constraints such as genomic architecture and secondary structure can and do influence codon usage in plant viruses, and likely in viruses of other hosts.

## 1. Introduction

All organisms exhibit some degree of codon usage bias (CUB), the unequal usage of synonymous codons [1,2]. Codon bias may vary among genes of the same organism, which is associated with factors like asymmetrical mutation pressures or tissue-specific gene expression, but is relatively uniform within the most highly expressed genes [3,4,5,6,7,8,9,10]. CUB is often explained as the product of two potentially competing factors: genomic base composition and translational selection (Table 1). In the absence of other mutational and selective pressures, CUB should result from the genomic frequency of A, C, G and T being reflected in third positions. When CUB diverges from the null hypothesis of genomic nucleotide content, translational selection—selection for optimal speed and accuracy of translation—is routinely invoked. Translational selection should exert an influence on CUB because preferred codons tend to correlate with the most common tRNAs [11,12], allowing for faster, yet accurate, codon recognition and translation of highly expressed genes [13,14]. However, genomic composition and translational selection need not be acting antagonistically on CUB, and sequences can show CUB distinct from that predicted by either force. 

When genomic base composition and known preferred codons correlate with observed CUB, both are potentially influencing CUB, and we cannot distinguish the relative strength of the forces. When observed CUB conflicts with the known preferred codons but adheres to genomic nucleotide content, the null hypothesis of overall base composition cannot be rejected, but translational selection can. Conversely, when preferred codons and observed CUB align, but CUB differs from that predicted by genomic base content, the null hypothesis can be rejected and translational selection deemed a more likely explanation. In the fourth case, neither overall base composition nor translational selection appears to be driving the observed CUB. This could be the result of direct conflict between the two forces yielding an intermediate state (i.e., the genome is enriched in adenine and suppresses cytosine, but the preferred codons tend to end in cytosine at the expense of NNA codons). Alternatively, another factor or factors must be influencing CUB. However, the vast majority of studies into the causes of CUB frame the question in terms of genomic base composition vs. translational selection, which precludes the consideration of additional important factors [15,16]. Considerations such as species-specific nmer promotion and suppression (*e.g.*, GATC for methyl-directed mismatch repair in *E.coli *[17], CpG in mammalian genomes [18]) are known to affect CUB, but are rarely considered in analyses of codon usage.

**Table 1:** Possible explanations for codon usage bias when the codon usage bias (CUB) of a gene of interest match or fail to match the genomic base composition and relative synonymous codon usage (RSCU) of a set of reference genes. For viruses, the comparison would be between the CUB of the viral genes and the CUB of their hosts.

		Base Composition	
		Match	No Match
RSCU	Match	Both?	Translational Selection
	No Match	Base Composition	Undetermined

This framework can be applied to studies of viral codon bias. Viruses with well characterized hosts are ideal systems in which to explore the forces shaping CUB because their genomic biases can be calculated from their viral genomes, but the hosts’ CUB reveal the translationally preferred codons. Viruses should experience translational selection to match the CUB of their hosts, as this should allow for faster translation of highly expressed viral genes, and consequently more rapid viral replication. It was recently documented that viruses with highly deoptimized CUB suffer a fitness cost [19]. However, surveys examining viral CUB have indicated that not all viruses are equally able to match their hosts’ codon preferences, and that this may be correlated with viral genomic architecture [20]. For instance, we previously demonstrated that double-stranded (dsDNA) coliphages were significantly better matched to *Escherichia coli*’s CUB than single-stranded (ssDNA) coliphages, because ssDNA phages had a preference for NNT codons, regardless of the hosts’ preferred codon usage [21]. 

To further investigate the evolutionary forces shaping viral codon usage, we investigated patterns of CUB in plant viruses with distinct genomic architectures. Plant viruses offer a unique opportunity to examine CUB because plant virus families often include members that only infect monocot hosts, and others that only infect eudicot hosts—two distinct translational environments. Monocots tend to have GC biased genes (53–56%), while eudicot genes generally have lower GC content (40-45%)[22]. Monocots exclusively prefer G- and C-ending codons, while eudicots prefer a combination of G- and T-ending codons in their most highly expressed genes (Table 2). These divergent hosts allow the strength of translational selection pressures to be compared among related viruses.

We analyzed three large groups of arthropod-vectored plant viruses: the positive sense ssRNA genus *Potyvirus* and family *Luteoviridae,* and the ssDNA family *Geminiviridae*. *Potyvirus* and *Geminiviridae* contain a comparable number of species with at least 15 sequences available for analysis (22 and 24, respectively). There were fewer appropriate *Luteoviridae* for analysis (8), but similar to the *Geminiviridae*, monocot- and dicot- infecting luteoviruses are organized into separate genera. These three groups differ in their genomic architectures: the filamentous potyviruses have a linear ~10kb genome that is expressed as a polyprotein, luteoviruses contain a linear genome of 5.3-5.7kb that is translated from subgenomic RNAs, and geminiviruses have one or two circular, ~2.7kb, ambisense genomic segments that are transcribed by host enzymes [23]. 

Unlike cellular organisms, which share related genes across extremely divergent clades, which can be used as the basis for phylogenies [24], very few functionally analogous viral genes are found in divergent taxa. We chose to examine the coat/capsid protein (CP) gene, a large ORF that is shared (though not homologous) among the three viral groups. While the CPs in some plant viruses serve the dual role of capsid and movement proteins [25], these factors only constrain amino acid usage, and should not impact synonymous codon usage. Similarly, the CPs of vectored viruses are under more strict selection against amino acid substitutions than those of non-vectored viruses [26], but as these arthropod-borne viruses are not expressing genes in the vector, it should not affect their codon bias. Therefore, analysis of CP genes best facilitates comparisons of results between the ssDNA and ssRNA viruses in this study. 

Additional analyses were required in *Geminiviridae*. The potyvirus CP and the luteovirus CP are each considered monophyletic; the monocot- and dicot- infecting viral sequences within each group once shared a common ancestral sequence. The monophyly of the CP in the *Geminiviridae* is assumed based on its unusual capsid shape [27], but the protein sequence of the ORF is highly divergent between begomoviruses and mastreviruses. Consequently, we also analyzed the CUB of the replication-associated gene (Rep), which is encoded in the complementary sense, for the geminiviruses. There is strong phylogenetic evidence for their Reps to be descended from a common ancestor [28,29]. While their CPs may be useful for comparisons to the RNA viruses, comparisons of the Rep CUB within *Geminiviridae* may be more appropriate, and comparable to analyses of the homologous CPs within each RNA virus family [27,30].

We compared the relative synonymous codon usage (RSCU [4]) of monocot- and eudicot-infecting members of each group to their hosts, to each other, and to viral genomic nucleotide composition to assess the relative importance of host codon preferences in viral CUB. Our results were surprisingly varied for viruses infecting common hosts, and demonstrate that pressures beyond base composition and translational selection affect CUB in ssDNA and ssRNA plant viruses.

## 2. Results

### 2.1. Monocots and Eudicots exhibit divergent CUB

**Figure 1:**
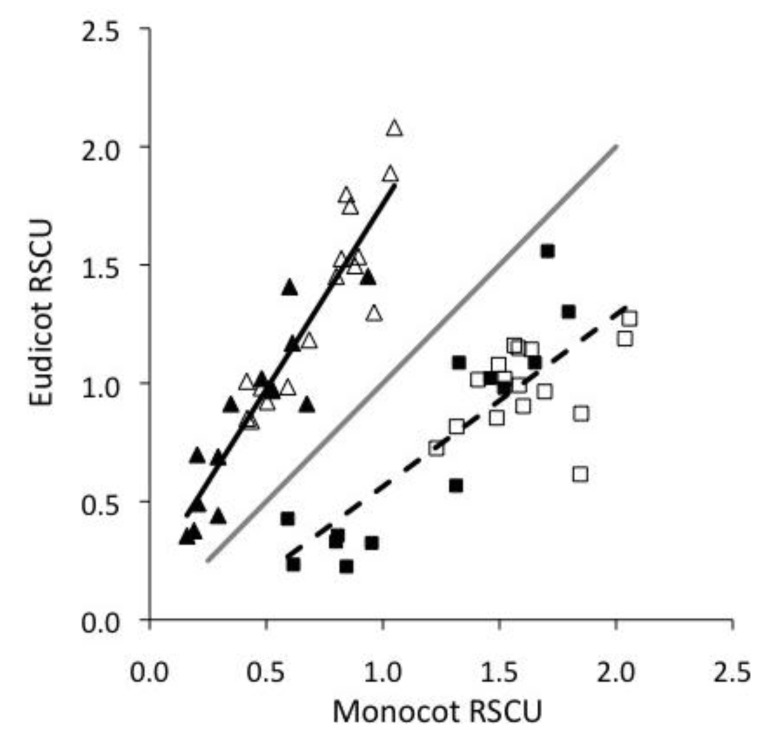
Relative synonymous codon usage (RSCU) correlation between monocot and eudicot highly expressed genes. Triangles represent A/T, squares are C/G, open symbols are pyrimidines, closed are purines. Solid line is best fit for A/T-ending codons (r=0.93), dashed is for G/C-ending (r=0.82). The grey line has a slope of 1 through the origin.

Plant codon preferences varied considerably between monocots and eudicots. The monocots analyzed exclusively preferred C- and G-ending codons in their highly expressed genes; fifteen of their overrepresented codons were C-ending, while the remaining five were G-ending (Table 2). These patterns were consistent with the codon preferences of all monocot genes [22]. Conversely, eudicots preferred a combination of NNT (six) and NNG (four) codons in their most highly expressed genes, in addition to two NNC codons (Table 2), which also agreed with their overall codon preferences. In all cases where plants preferred an NNG codon, no pyrimidines were possible in the third position (they were two-fold redundant amino acids, or the two-fold redundant portion of six-fold redundant amino acid codons). The RSCU of the highly expressed genes in the monocots and eudicots [22] were strongly correlated for A or T-ending codons (r=0.93), and for G or C-ending codons (r=0.82, Figure 1). However, best-fit lines for the two groups of codons both differ from a line with a slope of 1 through the origin, indicative of divergent codon preferences. Consequently, monocots and eudicots represent distinct translational environments, and should exert dissimilar translational selection pressures on the CUB of their respective viruses.

**Table 2:** Preferred codons in monocots and eudicots. Preferred codons are those with relative synonymous codon usage (RSCU) values that significantly exceed those of all synonymous codons (p<0.05, Bonferroni-corrected 2-tailed t-tests).

	Monocots				Eudicots		
	tac	aac	ccc		tac	aac	
	ctc	acc	gac				
NNC	atc	gcc	tgc				
	tcc	tac	cgc				
	agc	cac	ggc				
NNG	ttg	aag	cag		ttg	aag	cag
	gag	agg			gag		
NNT					ctt	tct	gct
					gtt	gat	cgt

### 2.2. Base composition does not explain most CUB in plant viruses

The potyviruses showed a consistent pattern of elevated adenine in their genomes, regardless of host, and also contained correspondingly lower levels of cytosine and guanine. The third position nucleotide content in CP genes differed significantly from that of the overall genome in every potyvirus we examined (chi-square tests, p<0.05). Luteoviruses showed consistent genomic base composition, having slightly elevated genomic adenine content, and relatively equitable use of cytosine, guanine, and thymine. Third position base frequencies were also consistent regardless of host, but differed significantly from genomic nucleotide composition in most luteoviruses. Third position base usage in two of the four eudicot-infecting luteoviruses did not differ significantly from genomic base content (chi-square tests, p>0.1). In the two remaining eudicot-infecting, and all four monocot-infecting luteoviruses, third positions diverged significantly from the genomes (chi-square tests, p<0.05). These findings indicate that genomic base composition is a poor predictor of CUB in luteoviruses and potyviruses.

Average third position base composition of CP genes in begomoviruses (dicot-infecting geminiviruses) and mastreviruses (monocot-infecting geminiviruses) also varied greatly from their respective genomic nucleotide contents (18 begomoviruses and 4 mastreviruses, chi-square tests, p<0.001). Base composition of synonymous sites in the Rep genes of all begomoviruses (n=14) and two out of three mastreviruses also diverged substantially from overall genomic nucleotide content (chi-square tests, p<0.05). As is the case for the ssRNA viruses, these results strongly suggest that genomic base composition does not drive CUB in geminiviruses.

### 2.3. RNA virus CUB is independent of host use

All potyviruses had somewhat similar codon preferences, independent of host: monocot- and eudicot-infecting potyviruses both generally preferred A- and T-ending codons (Table 3). Luteoviruses, both monocot- and eudicot-infecting, exhibited preferences for fewer codons overall, but tended to favor NNC codons. Despite this overall similarity they shared only two preferred codons (Table 3).

**Table 3:** Preferred codons in luteoviruses and potyviruses infecting monocot and eudicot hosts. Preferred codons are those with relative synonymous codon usage (RSCU) values that significantly exceed those of all other synonymous codons (p<0.05, Bonferroni-corrected 2-tailed t-tests).

		Potyviruses								Luteoviruses					
Host	monocot				eudicot				monocot				eudicot		
NNA	tca	cca	gca		tca	cca	gca						aga		
	aga	aaa	aca		aga	gaa	gga								
					caa										
NNC	tgc	cac							tgc	ttc	gac		tgc	ttc	gtc
									tac				atc	ctc	
NNT	tat	aat	gat		tat	aat	gat								
	ttt	ctt	gtt		ttt										
NNG					ttg				agg						

**Figure 2:**
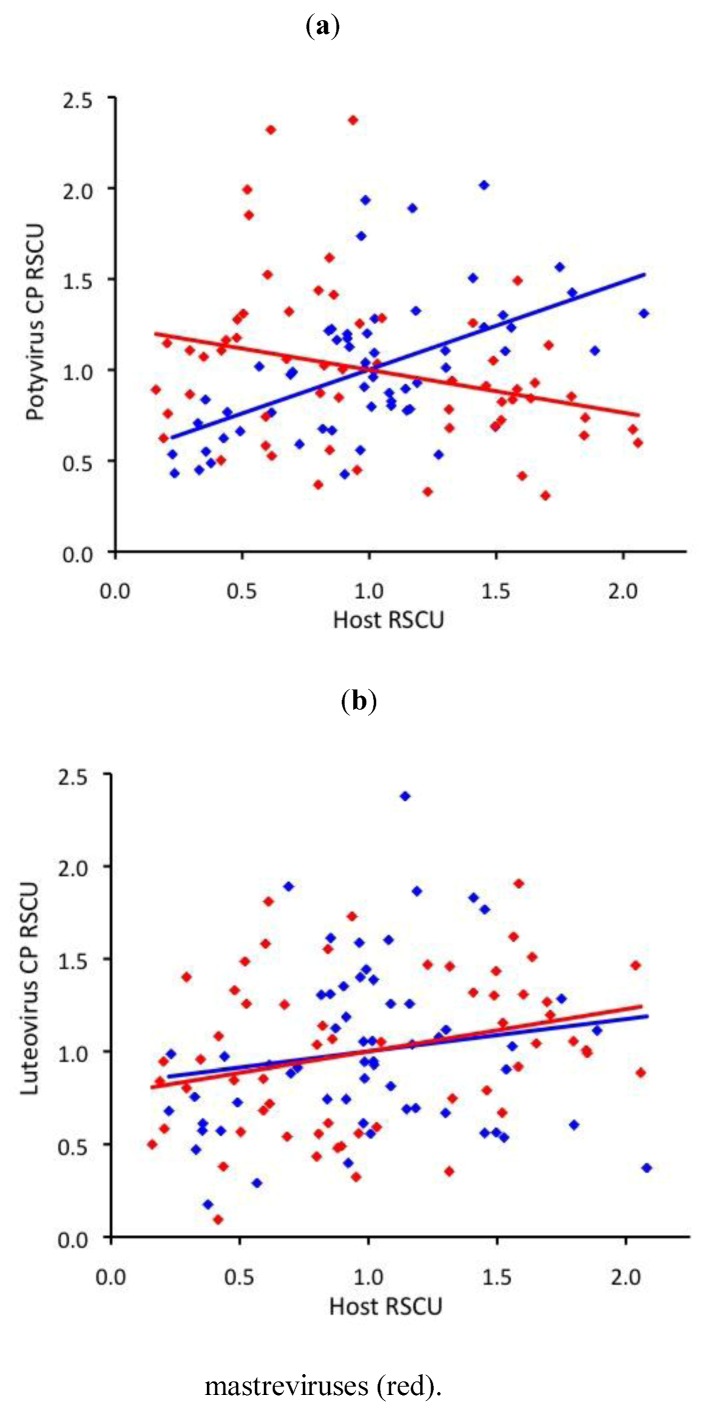
Correlation between host and virus coat/capsid protein (CP) relative synonymous codon usage (RSCU) for monocot-infecting (red) and eudicot-infecting (blue) **(a)** potyviruses and **(b)** luteoviruses.

Eudicot-infecting potyvirus RSCU was moderately correlated with eudicot RSCU (r=0.55), and monocot-infecting RSCU was actually weakly anti-correlated with monocot RSCU (r=-0.29, Figure 2a). Surprisingly, monocot-infecting potyvirus RSCU correlated with eudicot RSCU nearly as well as eudicot-infecting potyviruses (r=0.46). Monocot- and eudicot-infecting luteovirus RSCU weakly correlated with that of their hosts (r=0.29 and 0.16, respectively, Figure 2b). 

### 2.4. ssDNA virus RSCU does not indicate strong translational selection

ssDNA dicot-infecting begomovirus CP genes exhibited a strong preference for NNT codons, while begomovirus Rep sequences strongly favored NNA codons (Table 4). In the monocot-infecting mastreviruses, CP genes preferentially used C- and G-ending codons, but the Rep sequences did not exhibit a specific preference; overrepresented codons ended in all four bases (Table 4). Begomovirus genomes are ambisense; genes are encoded in the coding and complimentary sense [31]. The coding sequence of the Rep gene is complimented on the virion strand. As a consequence, third positions in this gene are present as the first base of anti-codons in the single-stranded viral genome. Therefore, these findings indicate begomovirus genomes are enriched for thymine at synonymous sites in both the CP ORF (with T-ending codons) and Rep ORF (with T-beginning anticodons).

**Table 4:** Preferred codons in mastrevirus and begomovirus Rep and CP genes. Preferred codons are those with relative synonymous codon usage (RSCU) values that significantly exceed those of all other synonymous codons (p<0.05, Bonferroni-corrected 2-tailed t-tests).

		Mastreviruses								Begomoviruses					
ORF	Rep				CP				Rep				CP		
NNA	aaa								aaa	caa	gga				
									aca	cca	gaa				
									aga						
NNC	tac				ttc	gcc	gac		ttc	ctc	tgc		ttc	ccc	
NNT	cat	cgt			agt				cat	aat	gat		cat	cgt	aat
													gat	act	tgt
													att	ggt	gtt
													tat		
NNG	ttg	agg			ttg	agg	gag		ttg				ttg	agg	gag
					aag	cag	ctg						aag		

**Figure 3:**
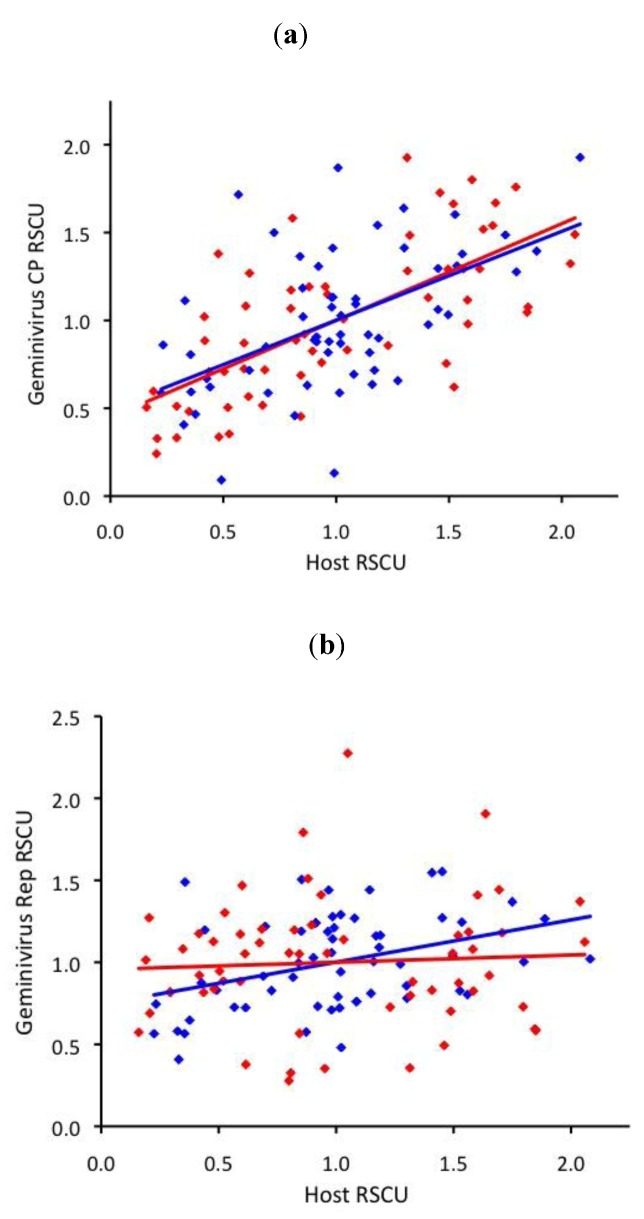
Correlation between relative synonymous codon usage (RSCU) of **(a)** geminivirus CP and **(b)** geminivirus Rep and host RSCU for eudicot-infecting begomoviruses (blue) and monocot-infecting mastreviruses (red).

In both begomoviruses and mastreviruses, the RSCU of their CP genes was moderately correlated to the RSCU of the highly expressed genes of their respective hosts (r=0.69 for begomoviruses, 0.53 for mastreviruses, Figure 3a). However, the Rep gene was not well correlated to their host RSCU in either begomoviruses (r=0.38) or mastreviruses (r=0.06, Figure 3b). Mastrevirus Rep RSCU actually matched that of eudicots better than the begomovirus Rep RSCU (r=0.71). 

**Figure 4:**
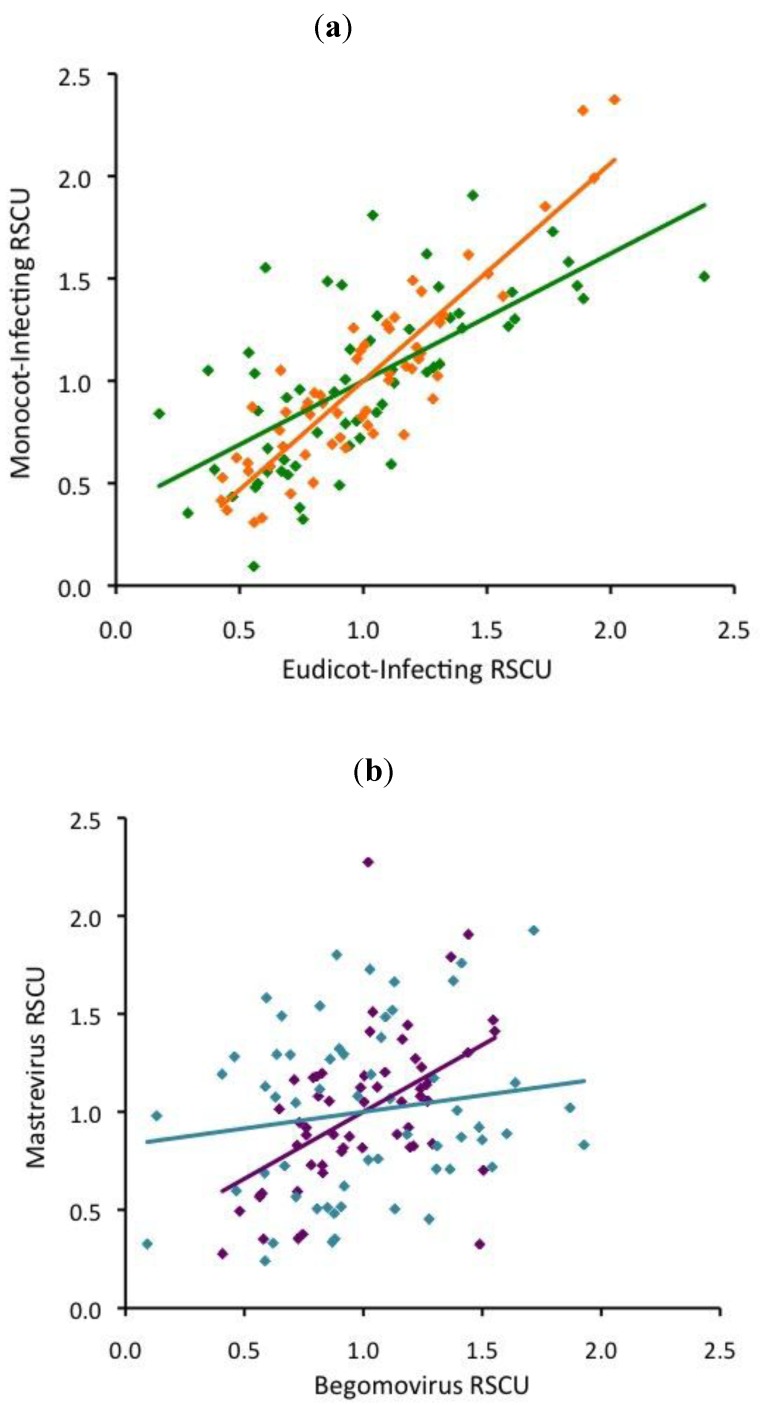
Correlation of relative synonymous codon usage (RSCU) between **(a)** monocot and eudicot-infecting potyvirus CPs (orange), luteovirus CPs (green) and **(b)** begomo- and mastrevirus CPs (aqua) and begomo- and mastrevirus Reps (purple).

The conservation of codon usage between monocot- and eudicot- infecting viruses within each group varied significantly. Potyvirus RSCUs were strongly correlated to each other (r=0.90), despite their hosts having divergent preferences (Figure 4a). Similar to the potyviruses, the two luteovirus groups exhibited a moderate correlation to each other (r=0.66, Figure 4a). Begomovirus and mastrevirus RSCUs were uncorrelated in the CP ORF (r=0.16), but moderately correlated in the Rep ORF (r=0.51, Figure 4b).

## 3. Discussion

### 3.1. Neither base composition nor translational selection explains our results

The null hypothesis, that synonymous codon usage is purely a function of the nucleotide base frequencies in the viral genome, was insufficient to explain codon preferences in all of the groups we examined. The discord between genomic frequencies and third position frequencies did not often manifest as more equitable nucleotide use in the third position; instead, codon use was more biased than genomic nucleotide frequencies in several cases (eudicot-infecting potyviruses, begomovirus CP). Similarly, the alternative hypothesis of translational selection failed to explain much of the plant virus CUB. Dicot-infecting potyviruses and geminivirus CP CUB were moderately correlated with their host CUB, but we did not find as high a correlation as in phage [21] or human RNA viruses [20].

The most common methods of assessing the relationship between genomic base composition and CUB involve using GC_3_, the GC content of the third codon position, as a measure of codon bias. GC_3_ is then compared to GC_1,2_, the GC content of the first and second codon positions, or ENC, the effective number of codons [32,33]. Both measures vary along predictable lines or curves when base composition drives CUB. By these measures, CUB in many viruses is strongly affected by overall base composition [33,34,35]. However, metrics that group AT and GC are unable to account for the over- or underrepresentation of a specific nucleotide in the third position at the expense of its complement, which is of special concern when analyzing CUB in single-stranded viruses. In the begomoviruses we analyzed, the average GC_3_ is approximately equal in the CP, Rep, and whole genome, but the CP (and genome) favor guanine while cytosine is overrepresented in the Rep. Consequently, our methods require a higher degree of agreement between the overall genomic base content and gene third position for base composition to be considered a plausible explanation for CUB.

Furthermore, many studies of viral CUB do not explicitly evaluate other factors that can drive codon bias. Rather, the null hypothesis is tested—does CUB follow the predicted relationship between GC_3_ and GC_1,2_ or ENC—and if not rejected, the effects of translational selection and other possible factors are not subsequently analyzed [15,16,34,36]. Other studies attribute the rejection to translational selection, but fail to consider additional factors [37,38,39]. 

### 3.2. Possible alternative explanation for CUB in plant viruses

Genes of propagative arthropod-vectored viruses (those that replicate within their vectors) should be under dual selective pressures to maximize replication speed within their plant hosts and their vectors. Consequently, vector codon preferences could influence codon bias in these viruses. However, the potyviruses and luteoviruses are nonpropagative [40], and while the evidence is more ambiguous in geminiviruses, they are generally considered nonpropagative as well [41,42], so translational selection is not acting on these viral genomes in their respective vectors. There is evidence of heightened purifying selection on capsid structure in vectored RNA viruses due to specific interactions between CP and vector [26], but in nonpropagative viruses, this pressure is independent of translation kinetics, instead acting solely on amino acid sequence. Therefore, translational selection within arthropod vectors cannot explain the observed CUB.

As potyviruses have high mutation rates [43], have diverged over at least thousands of years [44], and the different species analyzed were at least 25% divergent by nucleotide [23], it is impossible that the common synonymous codon usage we observed is an accident of recent fixation. It is similarly unlikely that a recent host-shift from eudicots (to which potyvirus CUB is better matched) brought recently diverged potyviruses into monocots [44]. Luteoviruses [45] and geminiviruses [46,47,48] evolve at similarly high speeds, so it is unlikely that these correlations are due to accidental historical contingency. It would further be expected that third positions would be saturated after thousands, if not millions of years of divergence [49]. Given these factors, it is most likely that the correlation among monocot- and eudicot-infecting members of each group is due to a similar set of pressures affecting CUB of each group as a whole. 

One possible factor that may influence codon bias in ssRNA viruses is selective constraints on secondary structure. ssRNA viral genomes often contain complex secondary structures that are important for replication or gene expression [50]. Disruption of these structures can inhibit one or both processes, reducing viral fitness. Substitutions are often observed in pairs: an initial mutation and a compensatory mutation that restores base pairing across stems in stem-loop structures, for instance [51]. These factors should manifest as more constrained codon usage at specific sites, though the effects on overall codon usage are ambiguous. 

The begomovirus CP, which strongly preferred NNT codons, aligned well with the preference of their eudicot hosts for T-ending codons and correlated strongly with host RSCU. Despite the significant differences between third position and genomic base content, these results also indicate the potential importance of the thymine enrichment of high-AT begomovirus genomes. Therefore, it is tempting to explain these data as the result of the combination of compositional constraints and strong translational selection, even if third position base use significantly differed from that of the entire genome. Conversely, begomovirus Rep sequences have different preferences and demand a different explanation. CUB is not explained by base composition, but the prevalence of A-ending codons and the weak correlation (r=0.37, Figure 4b) between host and virus RSCU suggests weak translational selection. It is unlikely that these two genes are subject to such divergent pressures that they would exhibit such inverse biases, as CUB tends to be similar within species [52]. However, neither base composition nor translational selection is sufficient to explain begomovirus CUB.

Similarly, CUB in the mastreviruses is not easily explained. In particular, the Rep has no well-defined codon preferences, which is not predicted by the genomic nucleotide composition, translational selection for their hosts’ CUB, nor an antagonistic relationship between the two. Consequently, these two factors alone are not sufficient to explain mastrevirus CUB.

The ssDNA architecture of geminiviruses provides a possible explanation for their CUB. ssDNA is prone to rapid cytosine deamination to uracil [53], and this process may explain the preference forT-ending codons in ssDNA phages, even in hosts with low AT% [21]. If this process also affects eukaryotic ssDNA viruses, we would expect a very different CUB profile compared to that which is determined by only base content and translational selection. Specifically, strong translational selection predicts uniform codon usage in both CP and Rep, but a strong, biased mutational pressure predicts the begomovirus preference for A-ending codons in the Rep sequences, given that they are encoded in the negative sense. C→T transitions may be tolerated only at synonymous sites, resulting in an overabundance of thymine in the genomic sequence, and a corresponding preference for adenine in the Rep coding sequence. When viewed as they are encoded in the genome, begomovirus CP and Rep nucleotide preferences at synonymous sites are remarkably consistent: both strongly prefer T-ending codons/T-beginning anticodons, suggesting that this biased mutational pressure may contribute to geminivirus CUB. A recent study of the ssDNA porcine circovirus also shows a preference for T-ending codons that differs from the codon preferences of their swine hosts, and is not due to genomic composition [54].

We believe it is very likely that a biased C→T mutational pressure affects eukaryotic ssDNA viruses. Eudicot-infecting begomoviruses are known to exhibit a long-term C→T substitution bias [46,47]. Additionally, ssDNA phages typically exhibit little secondary structure [55,56,57,58,59,60], and while very limited degrees have been documented in eukaryotic ssDNA viruses [61], it has not been observed in the ORFs we examined here. Consequently, these genomes are unconstrained by structural constraints at synonymous sites, and, because unpaired DNA is 100 times more susceptible to oxidative cytosine deamination to uracil than dsDNA [53], highly vulnerable to C→T transition. These oxidative deaminations are common in cellular genomes, but are efficiently repaired [62], while such changes in ssDNA viruses might simply go unrepaired [63]. Alternatively, host cytidine deaminases could be increasing viral thymine content by enzymatically deaminating cytosines. These enzymes are an innate mammalian anti-viral defense, and are active against both viral RNA and ssDNA [64]. Regardless of the exact mechanism, the evidence points to a biased mutational pressure at cytosines contributing to begomovirus evolution. 

Mastreviruses present a contrary case: whether or not they experience this potential thymine-enriching factor, their CUB remains unlikely in the absence of additional drivers. Mastreviruses may not experience the same mutational pressure from deamination, may have developed ways to compensate for it, or monocot hosts may interact with ssDNA genomes differently than eudicots. Neither their CP nor Rep sequences carry the signature of rapid deamination, and their CP CUB strongly adheres to host preferences, indicating the primacy of translational selection over base composition and other potential factors. Furthermore, an examination of maize streak virus revealed no evidence of the long-term C→T substitution bias evident in begomoviruses [48]. Finally, unlike most organisms that have been studied, MSV exhibits high degrees of variance in CUB between different genes, and the reasons for this variation are unclear [33]. A single recently discovered eudicot-infecting mastrevirus sequence [65] exhibited codon usage preferences that differ from monocots, eudicots, and the other viruses we examined. Additional analysis is required to more precisely determine the forces affecting CUB in mastreviruses.

## 4. Methods

### 4.1. Host codon usage bias

Codon preferences in highly expressed genes for five monocot and six eudicot plants were determined using the RSCU data from Wang and Roossinck [22]. Average monocot and eudicot RSCU was calculated for each codon, and preferred codons were defined by Bonferroni-corrected two-tailed t-tests (Microsoft Excel) of average RSCU for synonymous codons. Each set of redundant codons was analyzed individually; no comparisons were made between non-redundant codons. For these analyses, codons for the six-fold degenerate amino acids (L, R, S) were divided into two-fold and four-fold redundant groups, for which RSCU values were calculated independently. This was done because the two groups of codons for these amino acids differ at non-synonymous sites and are consequently recognized by different groups of tRNA species, making it inappropriate to treat them as a single set of redundant codons. 

### 4.2. Plant virus datasets

All available complete CP sequences of luteoviruses and potyviruses were collected from GenBank between March and May of 2012. Only species with 15 or more full CP gene sequences were analyzed, and sequences with ambiguous nucleotides were excluded. Complete CP and complete Rep gene sequences of Geminiviruses (monocot-infecting mastreviruses and dicot-infecting begomoviruses) were downloaded from GenBank between January and April of 2012. As with the ssRNA viruses, only species with at least 15 full gene sequences were analyzed, and sequences with ambiguous nucleotides were excluded. Consequently, some geminivirus species could be included in one of our analyses (CP analysis) but not the other (Rep analysis). In total, we analyzed 1285 geminivirus Rep gene sequences, 1481 geminivirus, 1210 potyvirus, and 315 luteovirus CP gene sequences (Supplemental File 1).

### 4.3. Base composition as a null hypothesis

All sequences were formatted for analysis using ReadSeq (http://www-bimas.cit.nih.gov/molbio/readseq). CAICal [66] was used to calculate the viral base composition. Reference sequences for each viral species were collected from GenBank on June 12, 2012. Sequences were formatted with ReadSeq, and CAICal was used to determine overall and site-specific base composition for each sequence. Observed third position nucleotide counts were averaged for each species. Expected third position nucleotide counts were computed for each gene/species combination we analyzed based on the genomic nucleotide frequencies of the species’ reference genome and the length of the ORF in the reference genome. Chi-square tests were used to evaluate the differences between these observed and expected counts, with three degrees of freedom (MS Excel). In total, sixty-seven chi-square tests were carried out: one on the CP gene of each potyvirus, luteovirus, and geminivirus we examined, and one on the Rep gene of each geminivirus we analyzed.

### 4.4. Plant virus codon usage biases

Viral RSCU calculations were as for the plant hosts. RSCU values were calculated for each sequence in each viral genus (in the case of the potyviruses, monocot-infecting and dicot-infecting). Then, mean RSCU values were calculated for each viral genus/group. Preferred codons were again defined by Bonferroni-corrected two-tailed t-tests of average RSCU for synonymous codons, and determined separately for monocot- and dicot-infecting members of each viral group. 

### 4.5. Comparison with host CUB

Average RSCU values for the monocot- and eudicot-infecting viruses of each viral group were compared to those of their respective hosts to determine the correlation between host and viral CUB. Average RSCU of monocot-infecting and dicot-infecting viruses within each group were also compared to each other to determine if the correlation among related viruses was stronger than the correlation to their respective hosts. RSCU between two groups was classified as uncorrelated (r<0.50), moderately correlated (0.50≤r<0.70), or strongly correlated (r≥0.70). Translational selection was rejected when host and virus RSCU were uncorrelated, but considered in cases of moderate or strong correlation. 

## 5. Conclusions

Codon usage bias is most often presented as the result of two competing forces: translational selection and genomic base composition. The methods most often used to evaluate it are sometimes sufficient to distinguish one of these factors from the other. However, in situations where neither factor appears significant, the available methods are of little use. Viral genomic nucleotide composition does not appear to be driving CUB in plant viruses, but there is only weak evidence of translational selection influencing CUB. Present methods are unable to explain plant virus CUB. Therefore, new ways of analyzing CUB and evaluating its likely determinants are required to more accurately parse the large amount of genomic data now available.

## Supplementary Files

Supplementary File 1 Supplemental Table (XLSX, 56 KB)
